# Near infrared light examination as part of the management of sporadic pancreatic head insulinoma: Case report

**DOI:** 10.1016/j.ijscr.2019.09.024

**Published:** 2019-09-24

**Authors:** Dan Petru Constantinescu, Mihaela Ioana Constantinescu, Razvan Alexandru Ciocan, Stefan Chiorescu, Miana Gabriela Pop, Daniela Simona Pintea, Valentin Muntean

**Affiliations:** Department of Surgery, Faculty of Medicine, University of Medicine and Pharmacy “Iuliu Hatieganu”, Cluj Napoca, Romania

**Keywords:** Insulinoma, Near infrared light, Indocyanine green, Pancreatic neuroendocrine tumors, Case report

## Abstract

**Introduction:**

We report the case of a 77-year-old female patient with the diagnosis of pancreatic head insulinoma, in whom we used near infrared light (NIR) to detect synchronous pancreatic tumors and potential secondary lymph node or liver involvement. The patient presented with hypoglycemia manifesting by lipothymia. With the diagnosis of secretory neuroendocrine tumor (insulinoma) of the pancreatic head, cephalic pancreatoduodenectomy with the preservation of the pylorus was performed after NIR visualization of the pancreatic tumor mass. At 6, 12, 18 months postoperatively, the patient no longer had hypoglycemia and her general state was good.

**Conclusion:**

NIR with indocyanine green (ICG) evidences pancreatic neuroendocrine tumors, as well as possible synchronous tumors and secondary lymph node or liver involvement.

## Highlights

• 77-Year-old female patient with the diagnosis of pancreatic head insulinoma.
• We used near infrared light to detect synchronous pancreatic tumors.
• Near infrared light was used to detect secondary lymph node, liver metastasis.
• Was perform cephalic pancreatoduodenectomy.
• Evolution was favorable.

## 1 Introduction

Pancreatic insulinomas are benign tumors in 90% of the cases. Sporadic insulinoma occurs in the pancreas in 98% of cases and is usually small (0.5 cm–3 cm), but may have malignant characteristics if its size exceeds 2.5 or 3 cm. Surgery is the only curative treatment. The examinations used to evidence the location of these neuroendocrine tumors preoperatively are somatostatin receptor scintigraphy, echoendoscopy, F dihydroxyphenylalanine (F-DOPA) PET CT. Somatostatin receptor scintigraphy (SRS) has a sensitivity of about 50–60% in detecting benign insulinomas. Echoendoscopy (EES) has an 86% (57–100%) rate of detection, while intraoperative ultrasound identifies 92% (84–100%) of all insulinomas. If the preoperative location of insulinoma is difficult, F dihydroxyphenylalanine (F-DOPA) PET CT is indicated. Another examination to detect secretory pancreatic neuroendocrine tumors (PanNET) is glucagon-like peptide-1 PET CT, which is useful particularly in MEN-1 (1). Intraoperatively, PanNET can be examined by palpation, intraoperative ultrasound (IOUS) or near infrared (NIR) light [1].

A 77-year-old female patient was treated in the Surgical Department of the Cluj-Napoca Clinical Emergency Hospital.

The aim of this case report is to demonstrate whether ICG administration and NIR examination in pancreatic insulinoma are beneficial for patients.

The work in this case has been reported in line with the SCARE criteria [2].

Surgical Clinic II, “Iuliu Hatieganu” UMF Cluj-Napoca, Romania.

## 2 Case report

Caucasian, female patient NM aged 77 years, from an urban area, retired, was admitted to the Surgical Clinic II by transfer from the Clinic of Diabetes Mellitus and Nutrition Diseases for repeated lipothymia, symptomatic hypoglycemia. The patient’s personal pathological history included antral gastritis, Helicobacter pylori infection and hypertriglyceridemia. She was on hypolipidemic medication and she had never smoked. Objective examination showed an overweight patient with a BMI of 28.3 kg/m^2^, dark circles under eyes, moderately altered general status, hot, dehydrated skin, murmur in the aortic focus, diminished vesicular murmur bilaterally and left basal crackles, kyphotic chest, painless abdomen on superficial and deep palpation.

The diagnosis of pancreatic insulinoma was confirmed by Whipple’s triad: low glycemia, hypoglycemia-associated symptoms and disappearance of symptomatology when glycemia returns to normal (ingestion of sugar).

**Fig. 1:**
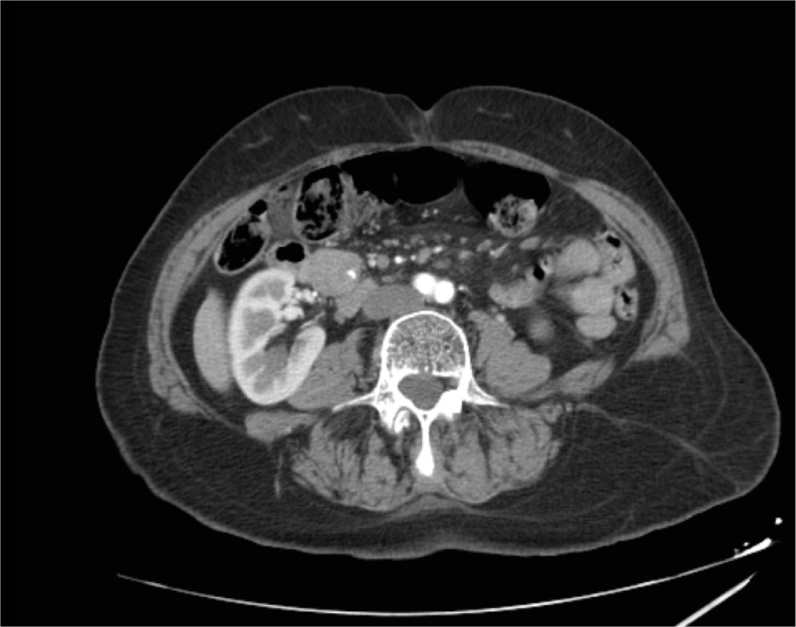
Arterial phase abdominal CT – tumor mass in the pancreatic uncinate process with inner calcification of 3.4/2 cm.

**Fig. 2:**
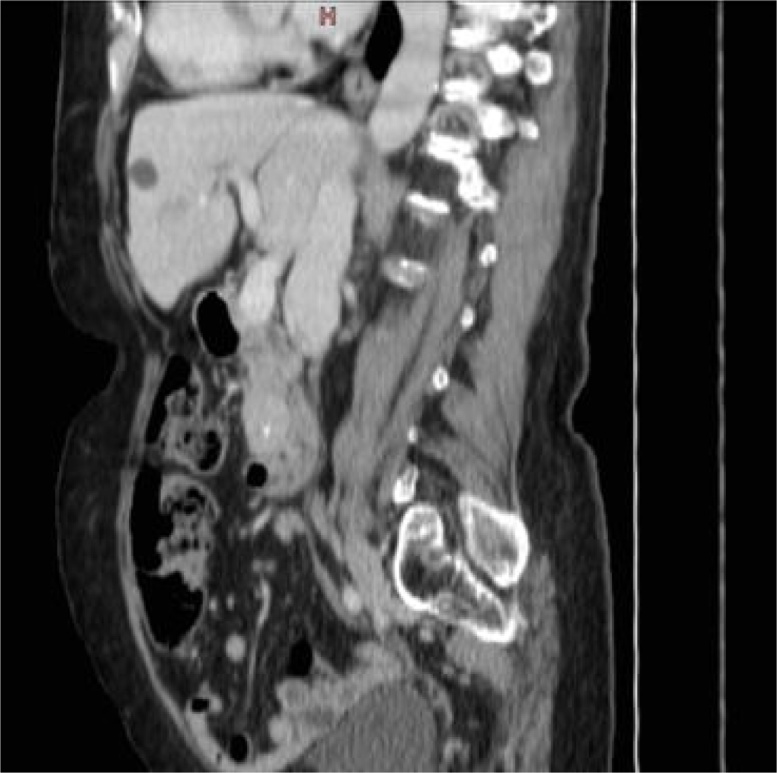
Abdominal CT, sagittal section: tumor mass of the pancreatic uncinate process.

Laboratory examinations showed glycemia 110 mg/dl under 20% glucose 3 vials 500 ml/day, glycosylated hemoglobin 4.37%, hypertriglyceridemia, hepatic and renal tests within normal limits, serum cortisol within normal limits, thyroid hormones within normal limits, parathormone within normal limits. Insulinemia in the hypoglycemic crisis was 28.7 μg/l (NV 2.6–24.9 μg/l). Imaging explorations (abdominopelvic CT with iv contrast, abdominal ultrasound) detected a hypoechogenic tumor mass in the pancreatic uncinate process, with gross calcification and peripheral vascularization 3.4/2 cm in diameter, without causing a mass effect in the pancreatic duct (Fig. 1, Fig. 2). Echoendoscopy-guided fine-needle aspiration puncture biopsy was performed. The histopathological result revealed moderately differentiated neuroendocrine pancreatic tumor (G2) (highly positive diffuse CD56), and Ki-67 was 6%.

The near infrared (NIR) detection device in the reported case was NIRF-800 probe, ArteMIS Handheld System, Quest Medical Imaging BV, Wieringerwerf, the Netherlands, which detects NIR between 790 nm and 805 nm, and indocyanine green (ICG) (25 mg/20 ml VERDYE). Five boluses of 2 ml from the 2.5 mg/ml ICG solution were administered.

*The exclusion criteria* (contraindications) *for* ICG administration in the *current case* were allergy to ICG and shellfish; hepatic or renal failure, hyperthyroidism, criteria that the patient denied. The patient’s informed consent for this procedure was obtained.

With the diagnosis of insulinoma of the pancreatic uncinate process, surgery was performed under general anesthesia. Intraoperative ultrasound evidenced a pancreatic uncinate process tumor of 3.3/2 cm, *which entered* into contact with the pancreatic duct without having a mass effect on it. No other pancreatic or hepatic tumor masses were ultrasonographically detected.

**Fig. 3:**
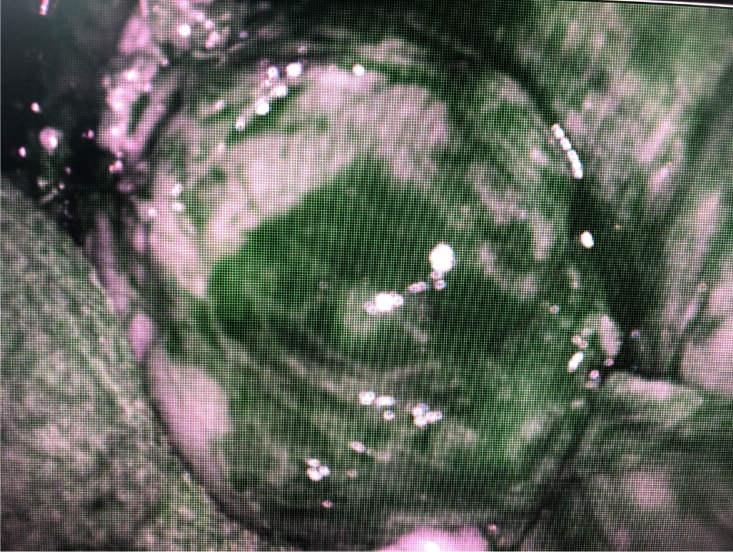
Tumor mass taking up ICG, situated in the pancreatic head.

**Fig. 4:**
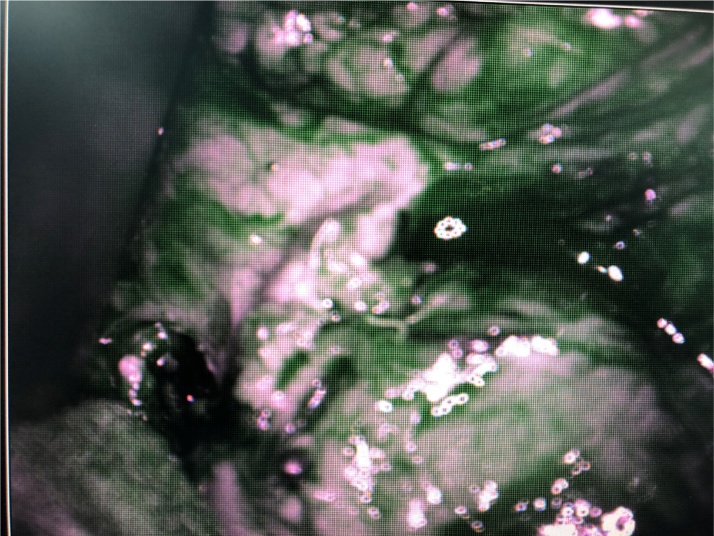
Exploration of the left pancreas with ICG and NIR.

*Intraoperatively*, after Kocher procedure, a tumor mass in the uncinate process was detected. To explore a possible multicentric insulinoma or secondary lymph node involvement in the hepatic hilum or liver metastases, ICG injection and examination with a NIR camera were carried out. No other tumor masses were found in the tail of the pancreas and no secondary hepatic or lymph node involvement was detected (Fig. 3, Fig. 4). With the diagnosis of secretory pancreatic neuroendocrine tumors (PanNET) located in the head of the pancreas in contact with the Wirsung duct, cephalic pancreatoduodenectomy with the preservation of the pylorus was performed. The operating time was 3 h; blood loss was 500 ml.

**Fig. 5:**
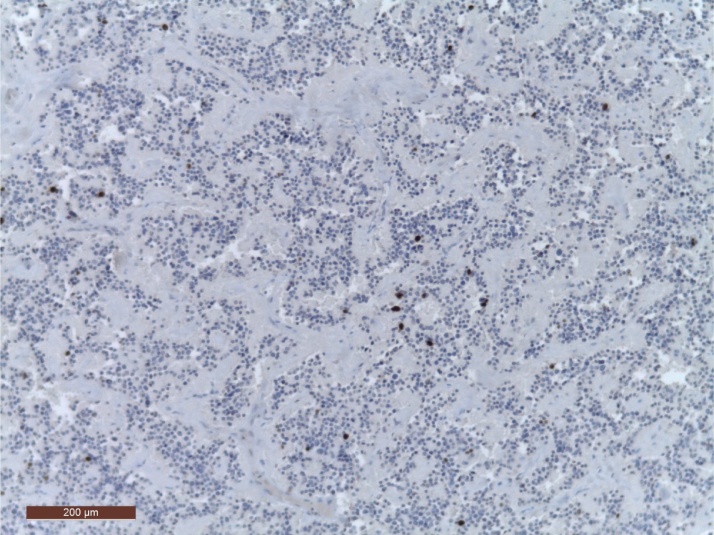
HE staining with Ki67 IHC.

Postoperative evolution was favorable (Clavien-Dindo grade I), the patient did not require 20% glucose intake, and discharge was on the 12th postoperative day. The histopathological result was G2 neuroendocrine tumor consistent in the clinical context with a pT2N0M0L0V0R0 insulinoma with a 3% Ki67 index (Fig. 5).

Evolution at 30 days was favorable, without ultrasonographically detected recurrences, and computed tomography with iv contrast detected no tumor recurrences and no secondary lymph node or liver involvement.

## 3 Discussions

The incidence of PanNET has increased over the last two decades [1].

Given the size of the tumor and its relationship with the pancreatic duct (the tumor was in direct connection with the main pancreatic duct), limited tumor resection could not be performed. The appropriateness of extended pancreatic resection was also supported by the high mitotic index (Ki67 3%).

### 3.1 Functional neuroendocrine tumors of the pancreas

In cases of **insulinomas**, surgery is the only curative treatment [4,5]. Because of their small size and low risk of malignancy, enucleation is the treatment of choice and this operation can be performed via laparoscopy if the location of the tumor is known and favorable (superficial lesion, located more than 2 or 3 mm from the main pancreatic duct (MPD) [5]. A formal resection pancreatoduodenectomy (PD), median or distal pancreatectomy can be necessary if enucleation is not possible because of the proximity of the lesion to the MPD or malignancy (10%) requiring lymphadenectomy (lymph node “picking” can be associated with enucleation). Preoperatively, important management elements include “patient education”, specifically frequent snacks and medical treatment to control hypoglycemia, whereas intravenous injection of glucose is usually not necessary. Diazoxide, a hyperglycemic sulfonamide (50–300 mg/day, a dose that can be increased up to 600 mg/day), is the most efficient treatment [5]. In case of undesirable effects or inefficacy, this drug can be associated with or replaced by others, in particular mTOR inhibitors (everolimus, rapamycin) [5].

Multiple endocrine neoplasia (MEN) should be excluded in the case of **gastrinoma** because, while long-term recovery is possible after R0 surgery for sporadic gastrinoma (20–45%), complete cure is very rare when gastrinoma is a component of MEN 1 (<10% when PD is not performed).

The **other** functional PanNET (glucagonoma, VIPoma, somatostatinoma, GRFoma, ACTHoma, PTH-rP secreting tumors or calcitoninoma) are even rarer. These tumors are usually voluminous, malignant, with locoregional or liver extension [5].

### 3.2 What type of pancreatic resection?

The type of surgery depends essentially on the size of the tumor and its location, but also on the relationship with nearby structures, the existence of synchronous lymph node metastases (LNM) or liver metastases (LM), patient co-morbidities, and possible hormonal secretion.

Pancreatoduodenectomy with tumor-free margins and lymphadenectomy is the carcinological operation for PanNET of the pancreatic head. The expected risk/benefit ratio has to be measured with extreme caution, particularly if the lesion is benign or of borderline malignancy [6]. This operation has at least three disadvantages. First, it is associated with the highest postoperative mortality among all pancreatic operations. Secondly, because of the volume of the resection, the patient is at risk for (mainly exocrine) pancreatic insufficiency [7]. Thirdly, this operation requires a bilioenteric anastomosis, which contraindicates or at least increases the risks of future procedures such as (chemo) embolization, or radiofrequency or microwave ablation of LM.

Distal pancreatectomy is the carcinological operation for lesions in the body or tail of the pancreas. This procedure, especially when performed for proximal lesions, removes the major part of the gland with a risk of pancreatic insufficiency (mainly endocrine) leading to de novo postoperative diabetes in about 15% of patients [8]. According to the characteristics of the PanNET (size of the lesion, locoregional involvement, tumor aggressiveness), the spleen can be preserved when its ablation is not indispensable for a R0 resection and/or lymphadenectomy. A tumor size greater than 3 cm seems to be a predictive factor for failure of preservation of the splenic vessels during PD [9].

In patients with a low carcinological risk, it is appropriate to preserve as much pancreatic parenchyma as possible, to avoid the risks of endocrine and/or exocrine insufficiency, particularly when the tumor is small, benign or of low-grade malignancy [10,11]. Enucleation can be performed via laparoscopy, with decreased duration of hospital stay, convalescence and abdominal parietal injury [13]. Because there is no tumor-free margin or routine lymphadenectomy, enucleation should be limited to benign or low-grade malignant tumors: insulinoma or small non-secretory PanNET (less than 2 or even 3 cm), without suspected lymph node or liver involvement on preoperative imaging. In case of isthmic or isthmo-corporeal PanNET, located to the left of the gastroduodenal artery, when enucleation is not possible, it is possible to perform a central pancreatectomy (CP) to avoid an extended left pancreatectomy [7].

In the absence of liver metastases, PanNET and metastatic lymph node resection is the only accepted curative treatment [3]. In a literature review, Jilesen et al. reported a 5-year mortality rate of 90% for completely resected, non-metastatic PanNET tumors [4].

Shirata et al. demonstrated that 100% of PanNET were visualized using fluorescence imaging [3].

Guarantying free resection margins and oncological radical resections, laparoscopy is becoming widely preferred over open surgery for the treatment of small benign or indeterminate pancreatic lesions, such as PanNET, the body–tail of the gland [6,14].

One of the most challenging aspects of the surgical plan is the exact localization of the tumors. Considering small PanNET, the reported preoperative and intraoperative detection rate is up to 92% [15], even when using palpation and IOUS, and it decreases to 50% for tumors below 1 cm. Even in high-volume centers, the intraoperative detection rate does not reach 100% [3].

Fluorescence-guided surgery has emerged as a novel intraoperative modality to assist surgeons to visualize tumors, sentinel lymph nodes, and vital structures in real time [11,12]. A vital dye, such as ICG administered iv, turns into fluorescent when excited by a light with specific wavelength, in the NIR spectrum.

Detection of PanNET with NIR prolongs surgery by approximately 8 min, but brings important benefits for visualizing the tumor [15].

Paiella et al. report a 0% reintervention and mortality rate in 10 cases operated by laparoscopy with a mean size of 2.4 cm (1–4 cm) and a Ki-67 index of 6% (2–12%). Nine patients underwent corporeo-caudal pancreatectomy and for one patient, tumor enucleation was performed. Eight PanNETs were non-secretory and two were insulinomas [15].

## 4 Conclusion

NIR by ICG administration is a method with almost 100% sensitivity and specificity for the intraoperative exploration of secretory PanNET and for the identification of other synchronous lesions, secondary lymph node involvement or liver metastases, which does not significantly prolong surgery.

## Sources of funding

No founding received.

## Ethical approval

Not applicable. The study is exempt from ethical approval in our institution.

## Consent

Consent has been obtained from the patient. No identifying details have been used in the article.

## Author’s contribution

V. Muntean: study concept, and final approval. V. Muntean, D. Constantinescu, R. Ciocan, D. Pintea treated the patient. D. Constantinescu, M. Constantinescu, S. Chiorescu wrote the first draft of the manuscript and the final draft of the manuscript, M. Pop photoediting, in editing manuscript. All authors read and approved the final manuscript.

## Registration of research studies

Not available.

## Guarantor

Valentin Muntean.

## Provenance and peer review

Not commissioned, externally peer-reviewed.

## Declaration of Competing Interest

None of the authors have any conflict of interest.
